# Barriers to the provision of non-communicable disease care in Zimbabwe: a qualitative study of primary health care nurses

**DOI:** 10.1186/s12912-022-00841-1

**Published:** 2022-03-18

**Authors:** Tiny Tinashe Kamvura, Jermaine M. Dambi, Ephraim Chiriseri, Jean Turner, Ruth Verhey, Dixon Chibanda

**Affiliations:** 1 The Friendship Bench, Research Support Centre, Faculty of Medicine and Health Sciences, University of Zimbabwe, Harare, Zimbabwe; 2 The Friendship Bench, Rehabilitation Sciences Unit, Faculty of Medicine and Health Sciences, University of Zimbabwe, Harare, Zimbabwe

**Keywords:** Barriers, Non-communicable diseases, Nurses, Primary care, Zimbabwe

## Abstract

**Background:**

Non-communicable diseases (NCDs) contribute significantly to the global disease burden, with low-and middle-income (LMICs) countries disproportionately affected. A significant knowledge gap in NCDs exacerbates the high burden, worsened by perennial health system challenges, including human and financial resources constraints. Primary health care workers play a crucial role in offering health care to most people in LMICs, and their views on the barriers to the provision of quality care for NCDs are critical. This study explored perceived barriers to providing NCDs care in primary health care facilities in Zimbabwe.

**Methods:**

In-depth, individual semi-structured interviews were conducted with general nurses in primary care facilities until data saturation was reached. We focused on diabetes, hypertension, and depression, the three most common conditions in primary care in Zimbabwe. We used thematic content analysis based on an interview guide developed following a situational analysis of NCDs care in Zimbabwe and views from patients with lived experiences.

**Results:**

Saturation was reached after interviewing 10 participants from five busy urban clinics. For all three NCDs, we identified four cross-cutting barriers, a) poor access to medication and functional equipment such as blood pressure machines, urinalysis strips; b) high cost of private care; c)poor working conditions; and d) poor awareness from both patients and the community which often resulted in the use of alternative potentially harmful remedies. Participants indicated that empowering communities could be an effective and low-cost approach to positive lifestyle changes and health-seeking behaviours. Participants indicated that the Friendship bench, a task-shifting programme working with trained community grandmothers, could provide a platform to introduce NCDs care at the community level. Also, creating community awareness and initiating screening at a community level through community health workers (CHWs) could reduce the workload on the clinic nursing staff.

**Conclusion:**

Our findings reflect those from other LMICs, with poor work conditions and resources shortages being salient barriers to optimal NCDs care at the facility level. Zimbabwe's primary health care system faces several challenges that call for exploring ways to alleviate worker fatigue through strengthened community-led care for NCDs. Empowering communities could improve awareness and positive lifestyle changes, thus optimising NCD care. Further, there is a need to optimise NCD care in urban Zimbabwe through a holistic and multisectoral approach to improve working conditions, basic clinical supplies and essential drugs, which are the significant challenges facing the country's health care sector. The Friendship Bench could be an ideal entry point for providing an integrated NCD care package for diabetes, hypertension and depression.

**Supplementary Information:**

The online version contains supplementary material available at 10.1186/s12912-022-00841-1.

## Background

Non-communicable diseases (NCDs) are a global public health concern; for example, NCDs account for 71% of deaths globally [1]. Low- and middle-income countries (LMICs) are disproportionally affected by the burden of NCDs; they account for an estimated 75% mortality rate and premature deaths globally [2, 3]. The Sub-Saharan Africa (SSA) region has been experiencing a surge in NCDs due to increases in cardiovascular risk factors such as; unhealthy diets, reduced physical activity, increased tobacco use, and the harmful use of alcohol [1, 4–7]. Consequently, NCDs are projected to be the leading cause of death and disability by 2030, eclipsing the burden of communicable diseases globally [1, 4, 7, 8]. Unfortunately, health systems in SSA are fragile, fragmented and under-resourced to address the increasing burden of NCDs [5, 9].

Exploring barriers to the delivery of care for NCDs by health workers in LMICs, a systematic review by Heller et al. [10] indicates that health care systems in LMICs are characterised by; excessive workload, poor staff retention, limited patient health literacy, absent data collection systems, medication and supply shortages, lack of monetary incentives and, underfunded health care structures [10]. Furthermore, SSA health systems are more oriented towards managing acute and episodic conditions than chronic conditions [11]. The integration of NCDs management into primary health care has also been affected by systematic institutional challenges, including poor policy implementation,, poor community engagement, low access to, and poorly integrated NCD care [12]. At the patient level, the utilisation of existing services is negatively affected by barriers such as; perceived risk, fear, lack of motivation, anxiety, affordability and hopelessness [11].

Access to knowledge about barriers and challenges related to NCDs care is severely limited by inadequate surveillance systems [13]. Available data on NCDs prevalence, the state of health care and the health care system is generally based on assumptions and generalisation from other settings [5, 9, 13]. For example, in Zimbabwe, existing knowledge on NCDs is not based on systematically collected data but instead on a combination of data from other African countries [6]. However, what is valid for one setting may not necessarily be true for another mainly because the nature of NCDs, their causes, consequences, and barriers to care are context-specific [14]. To address this challenge, researchers have stressed the need to generate contextually relevant data on NCDs in SSA countries that can help inform policy and practice, thus making it possible to develop effective, long-term health promotion strategies to help combat the NCD disease burden in the region [7, 13, 15].

The Friendship Bench (FB) is a brief psychological intervention delivered by trained community volunteers based on principles of cognitive behaviour therapy for common mental disorders [16, 17]. Anecdotal evidence from over 200 debrief sessions with grandmothers who deliver the programme shows that Friendship Bench has encountered clients with a combination of depression and comorbid hypertension and diabetes, hence the need to develop an integrated care package for comorbid NCDs. However, there is a paucity of knowledge on NCDs in Zimbabwe, particularly around the barriers to quality care. Therefore, we explored the perceptions and perspectives of registered general nurses (RGNs) to establish the current barriers to providing NCD care and management in urban primary health care clinics in Harare, Zimbabwe. In Zimbabwe, health care clinics are run mainly by nurses who refer severe cases to tertiary facilities per rising need [18]. The study also explored participants' perspectives regarding possible contextualised solutions. We also explored the Friendship Bench's potential role in achieving integrated care for NCDs, given the FB's extensive network of community-based CHWs. The findings from this study provided contextual information leading to the development of an integrated care package for diabetes, hypertension, and depression in primary care and community settings.

## Methods

### Research team

The interviews were conducted by five research assistants (RA) (three females and two males) who had a minimum of a bachelor's degree in Social Sciences. The RAs went through a five-day training workshop on qualitative methods, data collection and received continuous training after the initial training. The RAs were bilingual in Shona and English. All RAs were familiar with the key informants through prior data collection efforts. At the time of the study, the research team was working at the Friendship Bench.

### Study design

We used a descriptive qualitative study design to conduct in-depth semi-structured interviews with registered general nurses. The interviews focused on exploring nurses' experiences and perceptions of NCDs and their challenges in delivering NCD-focused care. The interviews focused on managing diabetes, hypertension,and depression; the three most common conditions at primary care in Zimbabwe. We developed an extensive interview guide based on the results of a situational analysis of the NCD care landscape in Zimbabwe that had been carried out earlier. In reporting this study, we have adhered to the COREQ (Consolidated criteria for reporting qualitative research) guidelines [19].

### Study setting

Study participants were drawn from five primary health care clinics (PHC) in Harare. The participants (nurses) have experience providing NCDs care, including diabetes, hypertension, and depression management. The five clinics were purposively selected from a pool of nine polyclinics that collectively reach over 600 000 households. There are two categories of primary care clinics in Harare, i.e. family health clinics and polyclinics. Family health clinics primarily provide maternity care, whilst polyclinics provide more comprehensive care, including NCD management. Study participants were nurses running the NCDs units at each clinic. No other individual was present when the interviews were conducted.

### Sampling and data collection

We used a purposeful sampling procedure by approaching the nurse in charge from the first clinic (C1). Afterwards, we asked the nurse in charge to recommend two staff members with extensive experience managing chronic conditions such as hypertension, diabetes, and depression. After completing the interview with the NCDs care experienced nurses, we asked them to recommend two colleagues with similar work experience from one of the other five PHCs. We carried out this snowball approach until we reached saturation. The total number of participants interviewed was 10. All invited participants agreed to be interviewed.

The semi-structured interview guides had a core list of open-ended questions and anticipated follow-up questions to help ensure that the researchers asked all participants a minimum set of identical questions to collect credible and comparable qualitative data (See Supplementary File 1). Interviews were conducted in English or Shona, based on the interviewee's preference. The interviewers conducted all interviews at the respective clinics that the interview participants worked. Each interview was digitally recorded, lasting an average of 45–60 min. Member checking was two days after the transcription was done with the interviewers restating and clarifying information that may not have been clear.

### Data analysis

Thematic content analysis was used to guide the analysis. Recorded interviews were transcribed verbatim and were appropriate, translated from Shona (the local language) to English immediately after each interview for analysis by experienced research assistants (RAs). Data analysis was done concurrently with data collection to monitor data saturation. The two senior researchers with extensive experience in qualitative analysis randomly selected two transcripts from each sample category and assigned codes that acted as labels for the ideas that appeared in the transcripts. After individual coding of all the transcripts, the researchers compared their findings to reach a consensus about a final code list that captured all identified ideas. After developing a single code list, all five RAs separately coded each of the transcripts, assigning codes from the code list to each instance of a key idea, and subsequently reconciled their assigned codes. All the team members reviewed the codes and then finalised the themes emerging from the data.

### Ethical issues

The study was approved by the Medical Research Council of Zimbabwe (Ref: MRCZ/A/2427). The Harare City Health Department granted permission and approval to conduct the study at the primary care clinics. Written informed consent was obtained from the participants before the interviews. Before conducting the interviews, the purpose of the study was explained to all the participants, and they were informed that they had the right to refuse to participate for any reason.

## Results

**Fig. 1:**
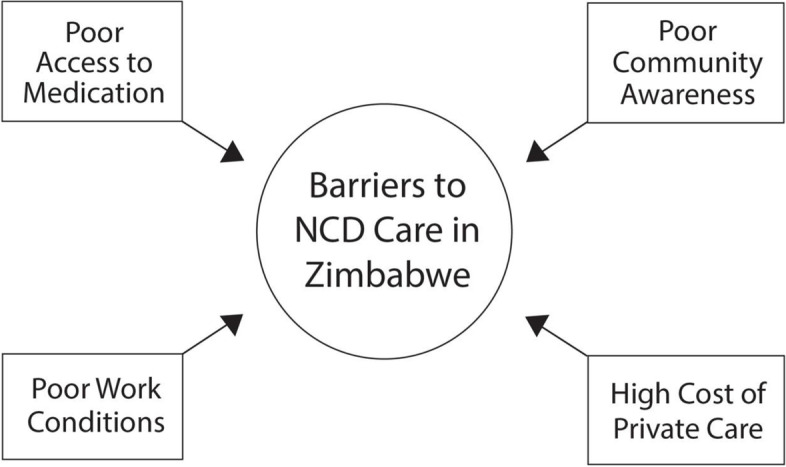
Barriers to NCD care in Zimbabwev. Abbreviation(s), NCD Non-communicable diseases

**Fig. 2:**
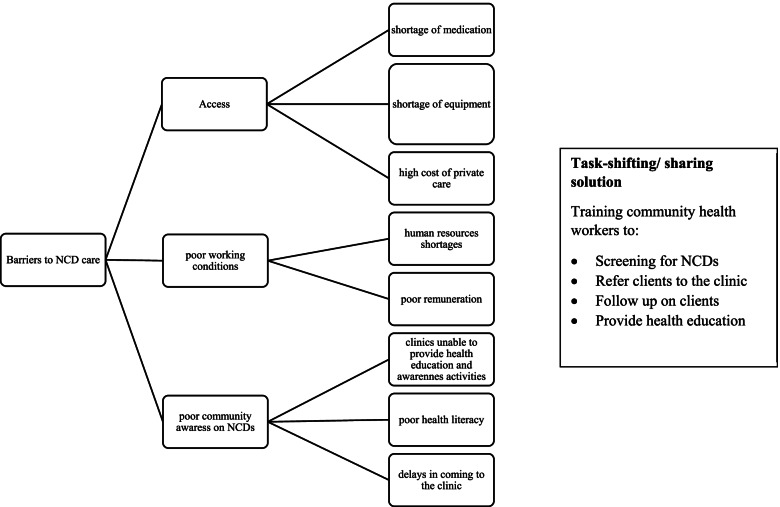
Major thematic codes generated from registered general nurse interviews. The coding tree above shows the barriers to NCD care within Harare urban primary care clinics as well as the potential solutions to the challenges being faced by the PHCs. Subordinate child codes elaborate the descriptions for each root code. All root codes and child codes were generated using thematic content analysis. Abbreviation(s): NCD Noncommunicable disease, PHC Primary health care clinics

Data saturation was reached after interviewing 10 participants. The participants were all female with a mean age of 43 and worked within primary care services for more than six (6) years; range four to twelve years. For all three NCDs (diabetes, hypertension and depression), we identified four barriers (Fig. 1) that were common across all five sites, i.e.; a) poor access to medication and functional equipment such as BP machines, urinalysis strips; b) high cost of private care resulting in no alternative options for communities; c) poor working conditions; and d) poor awareness on the 3 NCDs from both patients and the community which often resulted in the use of alternative potentially harmful remedies. A coding tree was designed show the root codes and their subordinate child codes (Fig. 2).

### ACCESS

#### Unavailability of medication

All five clinics pointed out that they were experiencing medication shortages for non-communicable conditions.

*P1: "medication like Enalapril (for hypertension) is unavailable most of the time. That's why sometimes when patients come, they do not get any medication, and that demoralises them, you know…."*

*P7: "There are some challenges there, to the point where there are times when the simplest drug, the most common drug like HCT (Hydrochlorothiazide) is out of stock. Like right now. It's currently out of stock. We don't have Metformin (diabetes). We do have Glibenclamide, but there are times when it is not available. Then there is medication like insulin for diabetes; we have not had those in years."*

*P8: Nifedipine is never available. Nifedipine and Enalapril come here and there. Mmm, which other medication is not always available? FD (depot injection for schizophrenia)."*

According to all participants, the unavailability of medication seemed to affect clinic user attendance rates. Nurses from clinics C3, C4, and C7 particularly emphasised how the medication shortage had a demoralising effect on patients.

*P4: "This lowers the patient's morale (when there is no medication) and causes them not to come to the clinic again. Your relationship with the patient also becomes strained."*

*P7: "We are failing to keep our patients with us because of the unavailability of medication. Even if they want to, so they feel they are running a loss. How can I just come for you to write (on my health card)".*

Other nurses highlighted that the medication shortage affected them personally and contributed to their collective sense of helplessness.

*P3: "It helps in treating diseases like severe depression. Because when there is no medication, the patients suffer a lot, and we become helpless as to how we could possibly help these people."*

*P5: "We hope we can get enough tools and enough medication. Because sometimes, when you talk to the patient, you end up feeling bad because you realise that there is no way this person can afford to buy medication. And you the nurse do not have anything to give. You cannot do anything; you just prescribe and leave it at that."*

### Shortage of equipment/machinery

The shortage of equipment/machinery for screening and testing purposes was another barrier participants highlighted as compromising their ability to do their work.

*P3: "We've still got a challenge in terms of the material resources. Like for diabetes patients, our glucometers are not functioning, but we need to monitor the blood levels……"*

*P1: "We have one functional blood pressure machine. But you find that it is used by multiple clinic departments…"*

*P8: "What I do not like about my job is that there is no adequate equipment. I could say we are improvising all the time."*

All participants pointed out that the shortage of equipment led to frequent disruptions in work flow.

*P3: "You know it demoralises us; for example, imagine yourself as a nurse moving across from one department to another just to get a blood pressure machine and come back. It demoralises you."*

### The high cost of private care

When the clinic does not have enough resources to help the patients with testing equipment and medication, they refer them to private institutions and pharmacies. However, private facilities are more expensive than PHC care. All the participants pointed out how the high cost of alternative services presented a challenge for the patients.

*P2: "As I said, when they (patients) come here at times, we have no option but to write a prescription and tell them to go and buy. But in terms of affordability …we hear them saying the prices are just unheard of! These guys from the pharmacy take advantage when they know that the medication is not available at the clinic."*

Participants five (5) and seven (7) pointed out that patients cannot afford to use private institutions for care as these were expensive for them.

*P5: "Umm, we do know how some of them cannot afford to buy food. Worse still to buy the medication. And worse still, to go and have my blood sugar checked at a private pharmacy."*

*P7: "If we manage to get the medication that is needed in every primary care clinic, it might help reduce the number of referrals for patients to buy medication at private pharmacies because some patients may not afford it."*

The shortage of adequate resources at local primary care clinics and the high cost of private care is making access to care difficult and created a double burden for patients.

### Poor work conditions

#### Lack of human resources

Human resource shortages were also an issue reported from all five clinics, posing a severe barrier to the delivery of quality care.

*P5: "I think the challenges are on the human resources. If we have an adequate workforce, I don't think there could be a challenge…."*

*PC7: "I think they should fix the issue of staff shortages so that our work goes on well and our patients get help in the shortest time."*

*P10: "Okay, this side we have staff shortages, the nurses are few. You saw me offloading things out there right, but I am not supposed to be doing that, but I do it because I only have one nurse available."*

The shortage of nurses causes delays for patients in getting help when they visit the clinic. Some of the nurses pointed out they were overwhelmed and felt frustrated with the poor working conditions.

*P2: "There are times when we get a lot of patients, and the nurses will be so few that we cannot even get to spend enough time with any of our patients."*

*P6: "Nowadays, they (patients) wait for quite a while because there is little staff. They are waiting for over 2 h before they are seen."*

#### Poor remuneration

The issue of salaries came up at all five clinics as the nurses highlighted that they were facing financial challenges because of low salaries.

*P4: "If it was possible, nurses should be given incentives so that we stay happy and we work happily. Even we are affected by these conditions that we are talking about. Depression and stuff. When I leave home, I leave problems there. I have trouble looking for cheap transport as I must hike a ride to work to save money for me to come to work every day. I do come to work, but my expectations as an employee are not being met."*

*P8: "With the way our economy is currently, I am not satisfied with my salary because it is not enough to travel to work, send children to school. Some of us now have children in tertiary education, some in secondary school and extended families. I have parents, I have a mother who needs to be taken care of. So, it's very difficult."*

Participants 2, 4, 8, and 10 pointed out that the issue of salaries also affects them psychologically and impacted negatively on their performance as health workers.

*P2: "… there is a shortage of staff because people are leaving for greener pastures and the issue of transport costs. The nurses themselves end up becoming depressed because of that. And when we are not mentally stable, we end up having lots of problems and a lot of challenges in delivering our duty."*

*P8: "l am not happy about the salaries. It is too little, it's causing stress and mental breakdown… as a sister in charge, I am a supervisor, as a supervisor I have to be well remunerated so that I do not borrow and to effectively discharge my work… If someone is well remunerated, they are motivated. So, the staff is not motivated because they are not well remunerated, hence some end up being rude."*

#### Poor community awareness of ncds

The unmet need for health education was a significant concern. All participants pointed to the need for health literacy for community members to improve health-seeking behaviour(s).

*P3: "What we can do to help people with hypertension and diabetes is to provide them with health education. For example, we can have community nurses who organise and conduct area health talks where they gather a lot of people, and they give them health education on conditions like hypertension and diabetes and encourage them to get screened."*

Although all clinics were aware of the value of sensitising communities on NCDs, they could not provide this as they were overwhelmed with running the understaffed clinics.

*P5:: "Yes, what is needed in the community is... yeah, we need to sensitise them in the community so that they know the signs and symptoms of everything. So that if they see a person with these signs and symptoms, they can come with the person to the clinic for further management*. *But we are too busy to go into the community."*

There was a strong belief from all five clinics that ignorance was a major factor at the community level.

*P1: "Obviously, it's ignorance of the people. They are not aware of these non-communicable diseases signs and symptoms presentations of these NCDs."*

*P8: "Give them enough information so that they know someone is being depressed or has diabetes, hypertension. These are some of the signs of depression….."*

Nurses reported that due to poor awareness, patients delayed coming to the clinic and only came when they were extremely sick, and their condition worsened, requiring referral to the tertiary facilities.

*P7: "Some patients come at a very later stage, years after having experienced symptoms related to diseases like diabetes, for example, because they did not know what it was and, as such. they would not seek help from the clinic or the doctor."*

Seven of the 10 participants pointed out that patients were using alternative sources of care such as traditional healers and local remedies due to poor awareness of NCDs.

*P5: "They believe that some of the signs and symptoms are due to them being bewitched, and they took traditional remedies whilst at home. And some of them, after talking to them, say: "I was once tested for BP, and it was raised, and I just ignored it. Or the doctor once initiated me on medication, but I just stopped because I thought I was well." Something like that. Some of them come only when they have a severe stroke."*

### Task-sharing/shifting as a potential solution

#### Training community health workers to provide basic NCDs care

When asked about how the Friendship Bench could potentially assist the clinic and the community regarding the conditions under study, participants proposed that existing Friendship Bench community health workers be trained to provide basic NCDs care. Through their health promotion activities, the community health workers were already providing NCDs care through following up on clinic patients and encouraging them to adhere to their medication.

*Int: "Alright, sister, what can be done with lay health workers to assist the treatment of people with these conditions?"*

*P1: "Firstly, looking at it we already work with the community health workers when it comes to hypertension and diabetes. I think the Friendship Bench and the Community Sister (nurse) can help them by providing them with more knowledge with regards to these conditions. I think they can be in a better position to help out more if they are provided with knowledge. They can be trained to provide health education either in Shona or English and also do follow-ups."*

Other nurses suggested that community health workers be trained to screen people for diabetes and hypertension. The screening could be done in the community to save patients the trouble of coming to the clinic every time they need their blood pressure or blood sugar levels checked.

*P7: "I think if community health workers are trained on how to test for diabetes using a glucometer it will help our client avoid frequent visit to the clinic. The clients will be tested by the community health workers in the community and if their blood sugar is high they can be referred to the clinic. Community health workers need to be trained on the blood pressure ranges, as in when it is normal or when it elevated so they can us check peoples blood pressures in the community."*

*P5: "You can try providing community health workers with screening machines that are user friendly and have a long battery life. You can also provide them with a manual that have the blood pressure and blood sugar levels, and information on when to refer people to the clinic. This will make their work much easier."*

The participants viewed task-shifting/ task-sharing as a way of mitigating the human resource shortage experienced in urban primary care facilities. The community health workers can be trained to screen people for NCDs, provide health education, and conduct follow-up visits. This work will have to take place directly in the community. However, the community health workers will have to be trained and provided with user-friendly screening machines and kits for this to be possible.

## Discussion

This study highlights the barriers faced by primary care nurses in providing adequate diabetes, hypertension and depression care. To the best of our knowledge, this is the first study of its kind exploring barriers within a Zimbabwean primary healthcare context. Similar findings have been highlighted in the region [20, 21]. Addressing poor access to care, limited resources, and the provision of public health knowledge and awareness are critical components of effective NCD care [22]. However, segmented service delivery remains a key challenge in most LMIC settings [23], with an absence of concerted involvement by governments, communities and healthcare providers in implementing integrated NCD care [24]. For the past four decades, healthcare systems in Sub-Sahara Africa have been hugely characterised by inadequate funding, underpaid staff, poorly maintained facilities and a shortage of core medical supplies and equipment [25].

As the burden of NCDs grows exponentially [26], there is concern that most primary health care settings are underequipped to manage this new epidemic [27]. There is also a need for effective policies to implement effective interventions, including allocating sufficient resources to fight NCDs [28]. The Addis Ababa Declaration states that countries must allocate at least 15% of their annual national budget to the health sector, which is believed to be adequate to revitalise health systems [29]. However, most LMICs are failing to reach that target and their health sectors are chronically underfunded. The Zimbabwean government for example, is said to only provides an estimated 21% of the country's required health finances, while the rest comes from donors, cooperation, and private health insurance [18]. Most of government health expenditure (65%) goes towards curative care, while prevention receives only 24%, and 10% goes to administration [18]. This is similar to what has been reported with regards to state of health care financing in LMICs [30, 31]. Healthcare financing literature argues that health financing systems in SSA are generally characterised by low government spending, underdeveloped health insurance schemes, and a high dependence on external/ donor funding[25]. On average less than 30% of the health expenditure in LMICs comes from the government compared to high-income countries (HICs) where governments fund close to 80% of their health expenditure [32]. This has resulted in developing countries having limited resources to ensure universal and equal access to health services [30–32]. In response to this, international banks and donor organisations have introduced mechanisms that are geared towards promoting NCD prevention and management efforts through bilateral and multilateral channels, for example the World Bank now provides NCD inclusive loans that are worth millions of dollars [33]. These funds are aimed at funding comprehensive primary health care efforts that incorporate health promotion, disease prevention, community engagement and measures to address the social determinant of health that are related to NCDs [34]. Improved health investment for NCDs' prevention can lead to better health outcomes with minimum funding for resource-constrained healthcare systems [35].

Findings from our study report a low availability of medical equipment and medication in primary health clinics in urban Zimbabwe to diagnose and manage NCDs. Literature shows similar trends in other low-income countries regarding the low availability of essential equipment and medication needed for the successful diagnoses and management of NCDs [36–38]. Sub Saharan Africa (SSA) is reported to have poor diagnoses rates for NCDs; for example, the Lancet Commission reported that more than half of hypertensive adults had not been diagnosed. Effective treatment coverage ranges from 1 to 31% among SSA countries [39]. Similar findings have been reported for diabetes, where only 22% of the population is reported to have been screened for diabetes, and approximately less than 25% have access to diabetes medication [36]. Consequently, this has resulted in patients opting for private health facilities and out-of-pocket payments, which are not affordable for most patients. Research shows that most SSA countries rely on out-of-pocket health care spending [25, 30, 32]. However, this presents a challenge for most people in low-income countries as out-of-pocket healthcare expenditure can surpass household income, further pushing them into poverty. Faced with the choice between health care and household necessities, some patients would either look for alternative sources of care that are less expensive or seek no treatment at all [30].

Participants for the current study also highlight that there is a general lack of knowledge and awareness on NCDs in the general population, resulting in adverse health outcomes. Existing literature states that a lack of awareness and knowledge about health conditions is a demand-side barrier that hinders the utilisation of health services [40]. The lack of knowledge about risk factors among the general population makes it challenging to reduce the burden of NCDs [41]. This is because people engage in NCD risk behaviours with a misunderstanding that they are engaging in healthy behaviour fuelled by lack of information on how to avoid the risks [42]. Kohori-Sewaga et al. (2020) show how people understand the importance of reducing salt intake and fatty foods but may not be aware of the recommended amounts of food intake they are encouraged to adhere to and how to put the knowledge in into practice [42]. This lack of awareness within communites may also inhibit the development of health services in communities. It limits the development of people's voices that can advocate for improving health services [40]. Scholars have proposed several solutions to tackle this, including engaging community health workers to provide health education on NCDs and embedding NCDs education within the school curriculum in secondary and tertiary education programs [40, 43, 44]. The multisectorial approach is presumed to increase the people quality of life, ensure planned urbanisation, and increased literacy on NCDs among LMICs [41].

In the current study, participants also reported that some patients were using alternative sources of care (i.e. traditional and faith healers) as they were more affordable than private health care. The use of alternative sources of care is viewed as a way of coping with the unavailability of medications at public health facilities and the high cost medications in private pharmacies [37]. Similar to the current study's findings existing literature also attributes this behaviour to low levels of health education and awareness among the general population [45]. Influenced by peers, family and tradition, some patients strongly believe in the effectiveness of herbal treatment and prefer herbs over prescribed medicine regardless of availability and access [46]. Traditional medicine is used as a supplement to conventional medicine [47]. Given the silent nature of NCDs, educating patients about their health conditions and their prescribed treatments is imperative to ensure positive health outcomes. Scholars recommend that future research be conducted to validate the efficacy of herbal medication and allopathy to generate empirical data about these herbs and their effect on health [46, 48]. This way, health providers can be better informed on how to recommend for or against the use of herbs and also, patients can be given accurate information [37, 48]. This is imperative given the increase in the practice of medical pluralism globally.

Our study reveals that the nursing staff were overstretched in all the five primary care clinics and unable to provide adequate care. While nursing staff receive support in managing people living with infectious diseases (e.g., HIV) from community health workers (CHWs) through home-based care and adherence counselling supported by non-governmental organisations (NGOs) working collaboratively with the Ministry of Health and Child Care (MoHCC) [49]; no structured support exists for NCDs care. Existing evidence suggests that, compared with standard care, engaging CHWs' support in health programmes can be effective in LMICs, particularly tobacco cessation, blood pressure, and diabetes control [50–52]. Integrating NCDs services within existing HIV and mental health programs have been suggested as a cost-effective way of increasing the availability, especially at the primary care level [36]. Patient driven interventions have also been reported to be effective in strengthening the quality of NCDs care [37]

In Zimbabwe, the Friendship Bench (FB) program has managed to show the effectiveness of engaging CHWs in the management of common mental disorders (CMD) such as depression and anxiety [53]. Although the CHWs offering the FB intervention have managed depression at the primary health care level, there has been little community focus. There has been no attempt to empower CHWs to manage patients with diabetes, hypertension and comorbid CMD. With limited resources and an overstretched staff complement at the PHC level, the need to task-shift care for all three NCDs is critical [36]. There is significant evidence supporting the cost-effectiveness of task-shifting in NCD care, resulting in health workforce strengthening in limited-resource settings [54, 55].

Through the Friendship Bench, over 600 CHWs have been trained to provide evidence-based care for CMD and improve access to care within primary health care facilities and communities. The Friendship Bench task-shifting model/approach based on stakeholder consensus through a theory of change workshops [56] has led to the development of a sustainable care package running for over ten years. With the limited resources unlikely to improve anytime soon in Zimbabwe, leveraging an existing programme such as the Friendship Bench could be an ideal entry point for the provision of an integrated NCD care package for diabetes, hypertension and depression, with the nursing staff referring mild to moderate cases to the Friendship Bench for support. According to the Lancet Taskforce on NCDs and economics, piggybacking on and working with existing infrastructures is an essential strategy, especially in settings where resources are limited [57]. There is, however, a need to involve critical stakeholders such as people living with diabetes, hypertension and depression through co-creation. Furthermore, there is also a need for continual lobbying policymakers to prioritise NCD management through a holistic and multisectoral approach. For example, improving working conditions, including basic clinical supplies and essential drugs, will optimise NCD care.

Our study suggests some simple steps which could be taken immediately. Creating community awareness and initiating screening at the community level through CHWs could reduce the workload on the clinic nursing staff. Indeed, based on our findings (Fig. 1), the existing CHWs involved in the Friendship Bench could be trained to provide basic screening and referral and support at the community level. We recently engaged key stakeholders through a theory of change workshop to explore the possibilities of such an approach (published elsewhere), and findings of the theory of change suggest that this would be feasible [58]. With NCDs estimated to overtake infectious diseases in LMIC in the next 20 years, task-shifting approaches similar to those successfully used in the fight against HIV [59] are promising in the care for NCDs.

## Conclusion

Our findings reflect those from neighbouring countries and LMICs, with poor work conditions and lack of appropriate equipment being the salient barriers to care for NCDs at the facility level. Zimbabwe's urban primary health care system faces several challenges that call for exploring ways to alleviate worker fatigue through strengthened community-led care for NCDs. Empowering communities could improve awareness and positive lifestyle changes, optimising NCD care. Also, there is a stern need for continual lobbying of policymakers to optimise NCD care within urban Zimbabwe through a holistic and multisectoral approach. It is essential to improve; working conditions, basic clinical supplies, and essential drugs availability; the country's significant health care sector challenges.

### Limitations

Our study limitations include the omission of people with lived experiences. However, findings from a separate study looking at the experience of people living with NCD are described in a separate study that supports a community focus with patient-centred care. While the engagement of CHWs is widely recognised, it remains unknown how they can be integrated into the care of multiple NCD conditions. We deliberately omitted this discussion because this is the next phase of our study, which will explore how an intervention can be developed.

## Supplementary Information

**Additional file 1.** Semi Structured Interview Guide for Nurses.

## Data Availability

The data that support the findings of this study are available from the corresponding authorupon reasonable request in a controlled access repository where relevant. The data are not publicly available due to them containing information that could compromise the participants consent and confidentiality.

## Abbreviations

BP: Blood Pressure
CBT: Cognitive Behavioural Therapy
CHW: Community health worker
CMD: Common Mental disorders
FB: Friendship Bench
FD: Fluphenazine Decanoate
HCT: Hydrochlorothiazide
HIV: Human Immune Virus
LMIC: Low- and Middle- Income Countries
MoHCC: Ministry of Health and Child Care
MRCZ: Medical Research Council of Zimbabwe
NCD: Non-communicable diseases
NIHR: National Institute for Health Research
PHC: Primary Health Care
RA: Research Assistants
RGN: Registered General Nurse
SDG: Sustainable development goals
SSA: Sub Saharan Africa
WHO: World Health Organization
