# Supplementary material for: Barriers to the provision of non-communicable disease care in Zimbabwe: a qualitative study of primary health care nurses
Source: BMC Nurs. 2022 Mar 18;21:64. doi: 10.1186/s12912-022-00841-1 (PMC8932172; doi:10.1186/s12912-022-00841-1)
Supplement: Supplementary file 1 — Additional file 1. Semi Structured Interview Guide for Nurses. [file 12912_2022_841_MOESM1_ESM.docx]

**Supplemental file 1: Semi Structured Interview Guide for Nurses**

**A. Participant Profile**

| 1 | Age | _______ years | |
| --- | --- | --- | --- |
| 2 | Gender | Male |  |
|  |  | Female |  |
| 3 | Professional training level | Nurse aid |  |
|  |  | Primary Care Nurse |  |
|  |  | Registered General Nurse |  |
|  |  | RGN Midwife |  |
| 4 | Work experience as nurse | < 5 years |  |
|  |  | 5-10 years |  |
|  |  | 11- 20 years |  |
|  |  | > 20 years |  |

**B. Situational Analysis**

5. How would you describe the mental health situation in your community?

1. Could you describe the mental health services that your patients need?
2. Have there been any changes in availability and/or accessibility to these services in the past 3 years?

a. What do you think are the drivers of these changes? (Probe: frequency and quality ofavailable trainings, community health networks, availability of medicines and supplies, sufficient staff, community demand for mental health services…)

**C. Impact of Friendship Bench**

1. Are you aware of the FB program? If yes, since when?

a. Please describe your involvement in the program? (Probe: referral to the Bench, community awareness, supervision of LHWs.)

b. How well does the FB intervention fit into the existing processes and practices in your setting?

c. In your opinion, is there a strong buy-in for this program from health care providers, LHWs and people in the community?

d. Have additional resources been deployed to implement the FB program? If so, how has this helped to improve the mental health situation in the community?

1. Do you have Circle KubatanaTose (CKT) meetings in your health facility? Could you describe how these groups are conducted?

**Needs of people with NCDs, mental health and substance use problems**

1. What do you think people with NCDs need when they come to the health facility?
2. What do you think patients want from the service? Anything else?
3. How satisfied do you think they are with the service?
4. In your view, what could improve the service at this health facility for people with NCDs?
5. What about in the community?
6. Are there actions that need to be taken in the community to help people with NCDs? Tell me about these?
7. How about people with depression? What do you think they want from the service here? Anything else? How satisfied do you think they are with the care?
8. In your view, what could improve the service at this health facility for people with depression, hypertension and diabetes?
9. What about in the community? Are there things that need to be done in the community to help people with depression, hypertension and diabetes?
10. Many people have more than one problem at the same time e.g. they could have diabetes and hypertension, or hypertension, and depression.
11. How well do you think comorbid conditions are detected in this health facility (i.e. when there are 2 or more health problems at the same time)?
12. How often do you screen for other (related) problems when a person is diagnosed as having an NCD or depression?
13. What would get in the way of screening for these problems routinely? What do you think about that?

**Care pathways and HMIS for people with NCDs, mental health**

1. How do people find out that they have an NCD (e.g. hypertension, diabetes, depression [probe separately]
2. How much delay is there for people with NCDs to come to the health facility? Why is there a delay?
3. When people come to the health facility, how are NCDs detected?
4. When an NCD, depression is detected at the health facility, what happens next?
5. What monitoring is in place to check whether or not the person is getting the correct care
6. How about changes in lifestyle e.g. diet, exercise, stopping substance use? How could patients be supported to make these changes?
7. How do you assess whether a person is responding properly to the treatment?

**E. Challenges and improvements**

1. What are the challenges that you and the LHWs are facing with regards to patient with depression, hypertension and diabetes?
2. What could be improved to make the your work more effective?
3. Are you aware of any individuals or groups in your community who do not have access to mental health services such as the Friendship Bench and NCDs treatment? If yes, how can their access be improved?
4. What recommendations do you have for the treatment, diagnosis of depression, hypertension and diabetes?
